# Patient-Generated Health Data in Pediatric Asthma: Exploratory Study of Providers' Information Needs

**DOI:** 10.2196/25413

**Published:** 2021-01-26

**Authors:** Victoria L Tiase, Katherine A Sward, Guilherme Del Fiol, Catherine Staes, Charlene Weir, Mollie R Cummins

**Affiliations:** 1 The Value Institute New York–Presbyterian Hospital New York, NY United States; 2 College of Nursing University of Utah Salt Lake City, UT United States; 3 Department of Biomedical Informatics University of Utah Salt Lake City, UT United States

**Keywords:** information needs, asthma, symptom management, mobile health, patient-generated health data, pediatrics, adolescents

## Abstract

**Background:**

Adolescents are using mobile health apps as a form of self-management to collect data on symptoms, medication adherence, and activity. Adding functionality to an electronic health record (EHR) to accommodate disease-specific patient-generated health data (PGHD) may support clinical care. However, little is known on how to incorporate PGHD in a way that informs care for patients. Pediatric asthma, a prevalent health issue in the United States with 6 million children diagnosed, serves as an exemplar condition to examine information needs related to PGHD.

**Objective:**

In this study we aimed to identify and prioritize asthma care tasks and decisions based on pediatric asthma guidelines and identify types of PGHD that might support the activities associated with the decisions. The purpose of this work is to provide guidance to mobile health app developers and EHR integration.

**Methods:**

We searched the literature for exemplar asthma mobile apps and examined the types of PGHD collected. We identified the information needs associated with each decision in accordance with consensus-based guidelines, assessed the suitability of PGHD to meet those needs, and validated our findings with expert asthma providers.

**Results:**

We mapped guideline-derived information needs to potential PGHD types and found PGHD that may be useful in meeting information needs. Information needs included types of symptoms, symptom triggers, medication adherence, and inhaler technique. Examples of suitable types of PGHD were Asthma Control Test calculations, exposures, and inhaler use. Providers suggested uncontrolled asthma as a place to focus PGHD efforts, indicating that they preferred to review PGHD at the time of the visit.

**Conclusions:**

We identified a manageable list of information requirements derived from clinical guidelines that can be used to guide the design and integration of PGHD into EHRs to support pediatric asthma management and advance mobile health app development. Mobile health app developers should examine PGHD information needs to inform EHR integration efforts.

## Introduction

### Background

Poorly controlled pediatric asthma continues to be a challenge. Pediatric asthma, the leading chronic disease among children, remains prevalent, and improvements in outcomes have stalled [1]. It is estimated that 6 million children under the age of 18 years in the United States have the chronic airway disease [2]. Despite evidence-based clinical guidelines, suboptimal treatment continues to contribute to a lack of asthma control [3]. Pediatric asthma can be managed with medications and trigger avoidance but requires continuous monitoring to assess control and detect triggers [1,4].

Understanding the complete picture of triggers and symptoms is essential for management, requiring health care providers to perform periodic assessments, adjust treatment plans, and personalize care [5]. However, a lack of objective data from patients means that a provider must depend on patient self-report, known to have reliability challenges, to make clinical decisions [6-8]. A potential solution may be the presentation of relevant patient-generated health data (PGHD) directly in the clinical documentation used by providers as they make decisions. PGHD such as biometric and physical activity, surveys, and health history are data captured electronically by patients outside of the clinic or hospital.

Mobile health (mHealth) technologies offer feasible opportunities to engage adolescents, persons between ages 10 to 19 years, in collecting PGHD [9]. Younger generations in every country are more likely than others to own a phone and are likely to use new technologies [8,10]. Moreover, adolescents engage with their mobile devices even while sick or hospitalized [11]. For pediatric asthma patients, mHealth apps support self-management, and wearable sensors provide ongoing monitoring capabilities [12,13]. The types of data collected from patients using smartphone asthma apps include symptoms, medication adherence, night awakenings, physical activity, and peak-flow expiratory rates [4,5,12,14]. Authors of two studies suggested that collecting the patient’s local environmental data, such as pollen counts, ambient temperature, and humidity, should also be considered [3,4]. When shared during clinical encounters, PGHD have the potential to facilitate assessment, diagnosis, and ongoing patient monitoring [15]. Presenting PGHD within the electronic health record (EHR) is envisioned as an optimal approach so that providers do not need to interrupt their cognitive processes and workflows to navigate between different systems.

Not much is known about which PGHD are of value or how to present PGHD to the providers in the EHR. A recent scoping review showed that EHR integration of PGHD is at an emergent phase; another identified only three asthma apps with the ability to share data with other apps [16,17]. Although many asthma apps exist, only a few of the mHealth technologies developed for childhood asthma have elicited feedback from clinicians [8], and even highly rated apps have not reported integration into clinical workflows [5]. The need for EHR data sharing has been recognized [15], and one study concluded that the introduction of smart-inhaler monitoring data into the EHR might support the development of individualized asthma treatment plans [7].

Despite the potential benefits, clinicians have expressed concerns that incorporating PGHD into the EHR will further contribute to information overload [13,18]. Additionally, studies reported issues with embedding mHealth technologies into clinical workflows and identified uncertainties about organizational readiness to integrate other data sources [13]. To ensure the clinical utility of PGHD, it is vital to understand the clinical workflows in which to integrate PGHD, as well as the specific tasks and decisions that PGHD must support and the relevant information needs of providers. Moreover, the discovery of information needs is necessary to inform future mHealth app implementations.

### Purpose

The purpose of this exploratory study was to identify and characterize a discrete set of tasks, decisions, and information needs of providers caring for patients with pediatric asthma and assess whether PGHD might provide useful information. We used outpatient care of patients with pediatric asthma as an exemplar clinical encounter where PGHD might have clinical value. By understanding these needs, we will be able to design interfaces and displays that optimally support the integration of PGHD into EHRs for the management of pediatric asthma.

## Methods

### Framework and Recruitment

We applied qualitative, descriptive methods to gain insights into key provider tasks, decisions, and information needs regarding PGHD and pediatric asthma. The procedures included analyzing published clinical guidelines to identify relevant decisions and consulting with providers treating patients with pediatric asthma to validate a discrete set of tasks and elicit their perspectives and priorities regarding the decisions, information needs, and potentially relevant PGHD.

We referred to the 3-phase model of needs assessment described by Altschuld and Kumar [19] as a guide. This model or framework proposes a practical process to assess needs that can be molded for a specific situation or setting. In the first phase of the model, preassessment, the goal is to determine what is already known regarding clinician needs and PGHD. We considered the preliminary examination of existing clinical guidelines as the activity to satisfy the preassessment phase or part 1 of this study. For the second phase or part 2 of the study, the assessment consisted of needs assessment procedures and data collection and validation with experts to move toward a full understanding. The third phase of the model, postassessment, involves the identification of strategies or development of solutions to meet the needs that were found during the assessment phase. We will consider the activities related to the postassessment phase in future research.

We recruited a convenience sample of three subject-matter experts (SMEs), domain specialists in pediatric asthma. Although no empirical evidence exists for the most appropriate number of experts for guideline review, similar studies that explored knowledge elicitation for consensus-based guidelines used at least three task experts [20,21]. We solicited SMEs by reputation using local clinical contacts. Inclusion criteria were the ability to read, understand, and use English as a primary language; self-reported expertise in pediatric asthma; and a history of medical practice in the United States. We excluded providers with adult-only asthma experience, and we did not compensate participants for their time. We received consent from all participants and obtained ethical approval for this study from the institutional review board of the University of Utah.

### Identify Tasks, Decisions, and Patient-Generated Health Data

We began with a review of the evidence-based pediatric asthma guidelines. We used the two main authoritative sources in pediatric asthma management from the National Institutes of Health National Heart, Lung, and Blood Institute (NHLBI) Expert Panel Report 3: Guidelines for the Diagnosis and Management of Asthma [22] and the Global Initiative for Asthma (GINA) Pocket Guide for Health Professionals [23]. The NHLBI asthma guidelines, in place for more than 25 years, focus on treatment protocols and monitoring for quality asthma care [3]. The GINA report serves as a practical tool to support asthma care and provides the basis for ongoing guideline revisions [24]. The development of these guidelines consisted of formal consensus methods commonly used for clinical guidelines.

According to the preassessment phase of the model, we conducted the needs assessment procedures and derived tasks, decisions, and information needs directly from the guidelines. In this context, a task is a professional duty or clinical responsibility related to patient care [25]. In the development of valid clinical guidelines, tasks are recommended to satisfy the goals of the guideline. Each of the tasks is linked to decisions: cognitive activities involving choices between alternatives or choices about what to believe or what to do [26]. In order to support the appropriate decision, information must be acquired from a person or an external system (an information need). One informatics expert on the research team extracted a list of high-level tasks from the GINA report and the primary task components from the NHLBI guidelines. Most of these were readily identified within each of the guideline documents, with tasks and decisions explicitly identified as such. Then, using the guidelines, the high-level decisions supported by each of the tasks were identified and listed alongside the information collected from the patient that assists with, or could assist with, making the decision. Once the extraction of tasks, decisions, and information needs was competed, the list was discussed with two other clinical informatics experts from the research team for agreement.

To further explain the extraction process, we used the assessment and monitoring task identified in the NHLBI guidelines as an example [22]. The assessment and monitoring task section of the guidelines identified two major decisions: assess the severity of the child’s asthma and decide the level of asthma control. The guidelines listed several information needs related to the decision for severity and control, such as frequency and intensity of the symptoms, functional limitations, exacerbations, lung function, and adverse effects from medication. The information needs were not labeled as such but were obvious from the text of the guidelines. Once we completed this exercise for all tasks from each of the guidelines, we synthesized the findings from both sources to create a single integrated set.

After we assembled the set of tasks, decisions, and information needs, we searched the literature for exemplar asthma mobile apps to assess whether PGHD might provide useful information. In January 2020, we searched PubMed using the terms *asthma mobile health applications* for studies that described asthma PGHD collection features. Given the small number of publications, we did not limit the search to pediatric-specific asthma apps. We examined the types of PGHD collected by each asthma mHealth app [7,14,17]. We inferred the ability of the discovered PGHD types to meet specific information needs by referring to the literature and using our clinical knowledge. Continuing from our previous example, one of the decisions for the assessment and monitoring task is to evaluate the level of asthma control. One asthma app collects the answers from the patient or caregiver and calculates an Asthma Control Test (ACT) score. The ACT is a well-validated, symptom-based tool used to assess symptom control that correlates clinically with specialist ratings and lung function [27]. The ACT is widely used and commonly part of strategies to stratify patients as having poorly controlled or well-controlled asthma [28].

We matched the discovered PGHD types to the corresponding information need in the integrated set. We continued with this process until we had a full set of mapped decisions, information needs, and PGHD for each major task category. All three clinical informatics experts from the research team reviewed the final set of tasks, decisions, information needs, and PGHD types and achieved consensus through discussion.

### Clinician Perspectives

We scheduled a 30-minute, in-person meeting with each SME independently to review the integrated set of tasks, decisions, information needs, and PGHD types. We also solicited general perceptions of the use of PGHD for adolescent asthma management. In a systematic fashion, we presented the SMEs with the mapped list and asked if it was the right list, if the items were in the order of importance for asthma treatment, and their general thoughts on using PGHD in practice.

We assessed the suitability of PGHD to support their information needs and generated field notes throughout the interview process. Based on the expert feedback, we created a final prioritized list of decisions, information needs, and PGHD types. We recorded participant responses as notes, examined the field notes for themes, and summarized responses. All three clinical informaticists reviewed the findings.

## Results

### Information Needs and Patient-Generated Health Data Types

Our analysis of the GINA report and NHLBI guidelines identified 4 high-level tasks:

- Assessment and monitoring
- Education for self-management in partnership with the patient and family
- Control of environmental factors and comorbid conditions
- Clinical management and pharmacotherapy

We found that many decisions corresponded to each of the tasks and that some decisions had multiple information needs. This analysis identified several key decisions needed to accomplish the 4 guideline-derived tasks. In our examination of exemplar mobile apps for asthma, we found 9 mHealth apps and 15 different PGHD types (Table 1). We matched the types of PGHD to the information needs derived from the guidelines (Table 2). However, we found that not all types of PGHD configured in the mHealth apps correspond directly with a guideline-derived information need.

**Table 1:** Asthma mobile health apps and patient-generated health data types.

App	Asthma action plan	ACT^a^	Journal	Activity level	Symptoms	Triggers	Medication reminders	Peak expiratory flow	Inhaler use	Medication use	Environmental factors	Survey data	Location	Mood
Asthma MD^b^	x		x		x	x	x	x						
Asthma Health App^c^				x	x	x				x		x	x	
Asthma Storylines^b^					x			x		x				x
Hailie^b^									x					
Kagen Air^b^	x				x	x	x			x	x		x	
Kiss My Asthma^b^	x				x									
My Asthma Pal^b^	x	x			x		x			x				
Smart Track^d^									x					
Propeller Health^b^									x	x	x			

^a^ACT: Asthma Control Test.

^b^Kagan and Garland [17].

^c^Genes et al [14].

^d^Chan et al [7].

**Table 2:** Types of asthma-relevant patient-generated health data.

Patient-generated health data	Information generated
Asthma Control Test	Symptom trajectory from the last 4 weeks
Exposures	Symptom triggers such as allergens, smoking, and perfume
Activity level	Level of physical activity
Symptoms	Type of symptoms and if daytime or nighttime
Peak-flow meter	Measurement of peak expiratory flow rates
Inhaler use	Medication adherence, last dose, missed doses
Asthma action plan	Progression toward goals and attitudes
Environmental factors	Pollen count and air quality
Concerns and/or questions	Ability to recognize worsening symptoms

### Perceptions About Patient-Generated Health Data and Pediatric Asthma Management

In August 2019, three primary care providers—two physicians and a nurse practitioner—participated in part 2. Based on their input, including their suggestions on importance to asthma treatment, we modified the initial guideline-derived list; the final list of high-priority information elements is provided in Table 3. The SMEs indicated that there were additional information needs related to triggers of asthma symptoms such as an insufficient level of dustproofing, pets, inadequate pest control measures, cleaning fluids, and other allergens; all of which may not be captured by PGHD. There was a specific interest in pollen, grass, pollution, and other environmental factors, and we added these triggers to the information needs of the decision point on determining exposure to risk factors. Although we identified decisions related to diagnosing in our initial integrated set, we excluded diagnostic decisions from our reviews with the providers based on our assumption that asthma-specific PGHD would be most useful for, and most likely collected by, children already diagnosed with asthma.

**Table 3:** Guideline-derived decisions and information needs with types of patient-generated health data.

Decision	Information needs	PGHD^a^
Determine level of symptom control	Symptom trajectory, types of symptoms, medication adherence, last dose, missed doses	Symptoms, ACT^b^, inhaler use
Determine exposure to risk factors	Symptom triggers such as allergens, smoking, pollen, poor air quality, perfume, inadequate dustproofing, pets, inadequate pest control measures, and cleaning fluids	Exposures, symptoms, environmental factors
Determine adjustments to medication regimen	Symptom trajectory, medication adherence, last dose, missed doses	Symptoms, inhaler use
Determine adjustments to action plan	Progression toward goals, attitudes, child’s and family’s ability to recognize worsening symptoms, level of physical activity	Asthma action plan, activity level
Determine ability to take medication	Observation of inhaler technique, medication adherence, last dose, missed doses	Inhaler use
Determine lung function	Lung function assessment, peak flow expiratory rates	Peak flow meter
Determine educational needs	Subjective questions or concerns from child or family	Concerns and/or questions

^a^PGHD: patient-generated health data.

^b^ACT: Asthma Control Test.

A few common perspectives resulted from the input of the SMEs. Each placed primary focus on uncontrolled asthma and indicated that PGHD would be most useful for adolescents, a subset of pediatric patients, who have trouble controlling their asthma. Perspectives concerning the timing for viewing PGHD were also prevalent. The SMEs expressed a desire to see the PGHD in the EHR at the time of the visit. They thought that it would be unusual to view the PGHD before a patient visit or between visits without an alert in the EHR or communication from the patient. Last, there was an interest in observing the inhaler technique and knowing whether the patients use spacers with their inhalers. The SMEs viewed proper inhaler technique as a critical component of medication adherence and pediatric asthma self-management. They thought that although patients might perform the proper inhaler technique during clinic visits, the technique might be inadequate outside of the visits.

## Discussion

### Principal Findings

A complete understanding of the information needs supported by PGHD is essential for the seamless integration of PGHD into workflows [13]. Identification of provider information needs is the first step in supporting the integration of PGHD into EHRs. In this study, we identified a list of high-priority decisions, information needs, and potential PGHD sources that can address the information needs of providers treating patients with pediatric asthma. We believe our findings demonstrate the suitability of PGHD to support clinical decisions for pediatric asthma. This work serves as a foundation to support future postassessment work such as evaluating the use of PGHD from mHealth apps and the integration of PGHD to EHRs.

In addition to the main findings, we uncovered aspects of PGHD use in pediatric asthma that may inform future research questions. Providers treating patients with pediatric asthma considered the use of PGHD to determine asthma triggers to be an essential part of treatment. Similar to other studies, providers expressed a need to know about general environmental factors, including air pollution and pollen levels [29,30]. We found that providers also wanted to learn about pest control (eg, roaches or rodents) in addition to contact with pets and other animals. Exposure products such as cleaning fluids, detergents, and perfumes were also of interest. Although it may prove difficult to capture all triggers using mobile technologies or sensors, the majority of asthma mHealth apps have the potential to include local air quality [17]. Further exploration is needed to fully understand the clinical utility of the inclusion of triggers in mHealth asthma apps.

The providers commented that inhaler use was an essential gap in their knowledge of patient behaviors, and in our review of the guidelines we found inhaler use to be a clear information need. Previous research reported that many patients use their inhaler poorly or share inhalers with friends or family members [1,8]. In our limited search, we found a lack of smart-inhaler mHealth apps that can capture and transmit data on inhaler technique or the use of spacers to providers. Technologies such as Respiro and Capmedic provide technique-related feedback to the user, but it is unclear whether providers can access technique assessment data [31,32]. As technologies advance, evidence related to the features of audio and video capture of inhaler use may be beneficial.

We found that providers were most interested in PGHD collected by patients whose asthma was severely uncontrolled. According to pediatric asthma guidelines, asthma severity is classified as mild, moderate, or severe, with severe asthma requiring the highest level of treatment [33]. Given the potential long-term repercussions, it is vital to treat children adequately in order to establish control early in life [34]. Although an emphasis on the PGHD of patients with severe asthma is reasonable, mHealth technologies identified gaps by noncompliant patients [8]. However, a study by Chan et al [7] reported that the use of mobile technologies for severe asthma might be most promising. As the number of mHealth apps increases, it may be worthwhile to collect additional evidence on the PGHD use—or lack of use—of pediatric mHealth apps for all types of asthma severity before focusing solely on uncontrolled cases.

Although particular care models determine the point in the care process when providers should review PGHD, we determined that in the context of outpatient pediatric asthma care, providers preferred to see the PGHD at the time of the visit. Other researchers have described programs that use nurse care coordinators or community health workers to review PGHD on a more ongoing basis [35,36]. Given that many patients with asthma have visits at the time of an exacerbation [1], it is reasonable that specialists in pediatric asthma see the most value for PGHD as part of their during-visit workflows [17].

In this study, providers expressed a keen interest in viewing PGHD directly from the EHR and not from another app. However, there is a risk that the potential richness of PGHD may get lost once it is added to an already complex and sometimes unsearchable EHR. Because of these comments, it would be worthwhile to investigate the requirements and provider preferences for the display of PGHD alongside EHR data and the locations in the EHR that may be most beneficial.

If the future of health care is personalization and individualized approaches to care, new strategies to harvest data from mobile technologies are needed [1]. As a first step, information technology specialists and health care providers should work together to determine clinical information needs for available PGHD and to update needs as new PGHD sources become available. There is great potential for PGHD to support the longitudinal care of patients with chronic disease. An understanding of the PGHD needs for pediatric asthma provides the opportunity to similarly explore the PGHD needs of other chronic diseases. The next step in the needs assessment framework (phase 3) indicates that actions must resolve the needs-based priorities [19]. We suggest a strategy for future research that examines the PGHD visualization and display preferences of providers to support the design of EHR integration.

### Limitations

Although this work was grounded in widely accepted guidelines, there may be nuances that were not accounted for, and all providers may not agree with or use the guidelines in practice. In addition, we validated our findings with primary care providers. It may be helpful to explore information needs with providers in other settings as needed.

### Conclusion

To optimally inform implementation approaches that integrate PGHD, the identification of provider information needs is essential. We extracted a set of tasks, decisions, and information needs derived from clinical guidelines and aligned them with PGHD types that may be collected by patients. By reviewing with providers caring for pediatric asthma patients in the outpatient setting, we validated the information needs and found that they align with some types of PGHD currently collected. This preliminary work serves to support the future design and development of mHealth apps and methods to integrate PGHD into EHRs that are in alignment with clinical information needs for chronic disease management.

## Abbreviations

ACT: Asthma Control Test
EHR: electronic health record
GINA: Global Initiative for Asthma
mHealth: mobile health
NHLBI: National Heart, Lung, and Blood Institute
PGHD: patient-generated health data
SME: subject-matter expert
